# The Impact of Chronic Obstructive Pulmonary Disease and Hospital Teaching Status on Mortality, Cost, and Length of Stay in Elective Total Hip Arthroplasty Patients

**DOI:** 10.7759/cureus.4443

**Published:** 2019-04-12

**Authors:** Cameron G Hanson, Kyle L Barner, Zakary Rose-Reneau, Michael Kortz

**Affiliations:** 1 Miscellaneous, Kansas City University of Medicine and Biosciences, Kansas City, USA; 2 Internal Medicine, Kansas City University of Medicine and Biosciences, Kansas City, USA; 3 Anatomy, Kansas City University of Medicine and Bioscience, Kansas City, USA

**Keywords:** copd, total hip arthroplasty, cost, mortality, length of stay, teaching status

## Abstract

Introduction

Total hip arthroplasty (THA) is a frequently performed surgery. Chronic obstructive pulmonary disease (COPD) is one of the most prevalent diseases in the United States and has been associated with higher complications in many orthopedic surgeries. The purpose of this study was to examine the clinical and economic impacts of COPD on the mortality, cost, and length of stay of those undergoing THA and the effect of hospital teaching status on these outcomes.

Methods

This retrospective cohort study identified adult patients (≥18 years) utilizing information from the Healthcare Cost and Utilization Program Nationwide Inpatient Sample (NIS) from 2012 to 2014 undergoing elective THA using ICD-9 codes. Patients missing key clinical identifiers or those who did not undergo THA were excluded. Mortality, cost, and length of stay were assessed. The COPD cohort was further analyzed by hospital teaching status, including urban teaching, urban non-teaching, and rural.

Results

An adjusted total of 7,652 patients with COPD and 768,000 patients without COPD undergoing THA were identified. COPD was associated with higher mortality rates, longer lengths of stay, and total charges. In the COPD cohort, teaching status did affect outcomes. Between urban teaching hospitals and urban non-teaching, chronic conditions were significantly higher in urban teaching hospitals, yet total charges were lower. LOS was longer in rural hospitals, however, all other variables, including costs, were not significantly different as compared to urban teaching hospitals. Between urban non-teaching hospitals and rural hospitals, the number of chronic conditions and LOS were higher in rural hospitals, yet costs were significantly less. Age and mortality rates were not significantly different between different teaching statuses.

Conclusion

COPD has a significant effect on mortality, length of stay, and cost in patients undergoing THA. Additionally, teaching status seems to play an interesting role in these variables. Preoperative planning may help surgeons mitigate some of these risks associated with COPD. Further work on how LOS and costs are optimized with regards to teaching status should be done.

## Introduction

In the United States, total hip arthroplasty (THA) is a commonly performed procedure in orthopedic surgery, most often in the treatment of osteoarthritis, osteoporosis, avascular necrosis of the femoral head, proximal femoral head fracture, or rheumatoid arthritis [1]. The average age for patients to undergo their first primary THA is 66.6 years (SD=10.2) [1]. The number of total hip arthroplasties performed in the United States for patients aged over 45 increased from 138,700, with a rate of 142.2 performed per 100,000 people in the year 2000, to 310,800 with a rate of 257 performed per 100,000 people in the year 2010 [2]. This trend is expected to continue in the near future, with an estimated 572,000 THAs performed by the year 2030 [3]. Regardless of its invasive nature, in general, THA is well-tolerated with relatively good outcomes [4].

Chronic obstructive pulmonary disease (COPD) is a common comorbidity with a prevalence of up to 12% of the population in key states and a national mortality rate of 39.1 per 100,000 in the United States [5]. COPD is the fourth leading cause of death in the United States and direct expenses related to the disease’s treatment are expected to rise to $49 billion by the year 2020 [6]. COPD care has previously been associated with anesthesia-related complications postoperatively and intraoperative or postoperative pulmonary complications [7]. It has also been shown to negatively impact outcomes in total hip arthroplasty, including 90-day mortality, one-year mortality, one-year wound infection, 30-day readmission for hospitalization, 30-day pneumonia, 30-day acute respiratory failure, 30-day cerebrovascular accident, and length of stay during hospitalization [8].

Teaching status in orthopedic surgery has also been shown to influence outcomes in THA [9]. However, among the studies that have examined the effect of COPD and orthopedic surgeries, the authors were unable to find any that included hospital teaching status as a tested variable. The purpose of this study was to examine the effect of hospital teaching status on procedure and care cost, hospital length of stay (LOS), mortality, and the number of chronic conditions in those undergoing elective THA with and without COPD.

## Materials and methods

Database

The National Inpatient Sample (NIS) is the largest, all-payer, publicly available inpatient care database in the United States and is part of the Healthcare Cost and Utilization Project (HCUP). It is a stratified 20% sample of all patient discharges from HCUP-participating academic and non-academic hospitals in 38 states and contains information on over seven million hospital discharges per year. The NIS has been shown to be adequate for the estimation of data that is nationally representative [10].

Sample selection

Deidentified patient discharge data from 2012-2014 were acquired from the NIS database and adult patients (≥18 years) undergoing elective total hip arthroplasty were identified using International Classification of Disease 9th edition (ICD-9) procedure code(s). Patients with a principal procedure code of total hip arthroplasty (International Classification of Diseases, Ninth Revision, procedure 81.51) were identified. From this sample, patients with COPD ICD-9 codes, including chronic bronchitis (491) or emphysema (492), along with their secondary codes, were extracted as described in Table 1.

**Table 1:** ICD 9th Edition COPD Codes ICD: International Classification of Diseases, COPD: Chronic Obstructive Pulmonary Disease

ICD-9 Code	ICD-9 Secondary Code
Chronic Bronchitis (491)	Simple chronic bronchitis (491.0), Mucopurulent chronic bronchitis (491.1), Obstructive chronic bronchitis w/o exac (491.20), Obstructive chronic bronchitis w(ac) exac (491.21), Obstructive chronic bronchitis w ac bronc (491.22), Chronic bronchitis NEC (491.8), Chronic bronchitis NOS (491.9)
Emphysema (492)	Emphysematous bleb (492.0), Emphysema NEC (492.8)

This subset of patients was then stratified by the teaching status of the hospital, including urban teaching, urban non-teaching, and rural. For this study, a hospital was considered a teaching hospital if it had one or more Accreditation Council for Graduate Medical Education (ACGME)-approved residency programs, was a member of the Council of Teaching Hospitals (COTH), or had a ratio of full-time equivalent interns and residents to beds of 0.25 or higher. Rural hospitals were not split according to teaching status, as rural teaching hospitals were rare [11]. Demographic and outcome data were extracted, including age at admission (years), length of hospital stay (days), total hospital charges (US dollars), number of chronic conditions, and mortality during hospitalization.

Data analysis

Data analysis was performed by one-way analysis of variance (ANOVA) with linear regression and chi-squared for significance values using SPSS software (IBM Corp., Armonk, NY, US). As the NIS samples constituted approximately 20% of United States hospital discharges, the results were multiplied by five in order to accurately represent the total population in the data. Ethical clearance and patient consent were not sought, as the NIS-HCUP database contains deidentified patient data. This study was determined to be Institutional Review Board (IRB) exempt. The statistical level of significance was decided at 𝛂=0.05.

## Results

A total of 7,985 patients with COPD and 768,000 patients without COPD undergoing elective THA were identified. 

COPD vs. non-COPD

Among all hospitals, COPD is associated with more costs, higher ages of admission, longer lengths of stay, and increased mortality when compared to patients who do not have COPD undergoing elective THA (Table 2). The average age for COPD patients who underwent elective THA was 68.24 years old (standard deviation (SD) of 10.8, 95% confidence interval (CI) ± 0.24). The average age for a non-COPD patient who underwent an elective THA was 64.92 years old (SD of 11.9, 95% CI ± 0.12). The average cost of a COPD patient was $65,987 (SD of $28,470, 95% CI ± $634), and the average cost of a non-COPD patient was $55,585 (SD $36,560, 95% CI ± $82).

**Table 2:** COPD vs. non-COPD COPD: Chronic Obstructive Pulmonary Disease

Characteristic	COPD (n=7,985)	Non-COPD (n=768,000)	Significance Level
Admission Age (years)	68.24	64.92	p<0.001
Cost (U.S. Dollars)	$65,978	$55,585	p<0.001
Length of Stay (Days)	3.49	2.82	p<0.001
Mortality	0.7%	0.1%	p<0.001

COPD and teaching status

The analysis of the impact of COPD on THA with regards to teaching status yielded unexpected results (Table 3). Overall, the mortality rate was not significantly different between different teaching statuses (p>0.5). From the data, most COPD patients undergoing elective THA had it performed at an urban teaching hospital (n=4,280). The average age of admission for urban teaching, urban non-teaching, and rural hospitals was 65.7 years (SD 10.6, 95% CI ± 2.1), 68.2 years old (SD 10.2, 95% CI ± 0.2), and 67.2 years old (SD 11.1, 95% CI ± 0.8), respectively. The average inpatient cost was $59,174 (SD $38,172, 95% CI ± $1,144), $63,827 (SD $33,280, 95% CI ± $1,201), and $56,141 (SD $37,339, 95% CI ± $2,664). The average length of stay was 3.47 days (SD 2.18, 95% CI ± 0.07) for urban teaching, 3.37 days (SD 2.37, 95% CI ± 1.09) for urban non-teaching, and 3.97 days (SD 2.97, 95% CI ± 0.21) for rural. The average number of chronic conditions was 7.58 (SD 2.86, 95% CI ± 0.09) for urban teaching, 7.07 (SD 2.75, 95% CI ± 0.10) for urban non-teaching, and 7.64 (SD 2.97, 95% CI ± 0.22) for rural.

**Table 3:** COPD and Teaching Status COPD: Chronic Obstructive Pulmonary Disease

Characteristic	Urban Teaching (n=4280)	Urban Non-Teaching (n=2950)	Rural (n=755)
Admission Age (years)	67.53	68.20	67.28
Cost (U.S. dollars)	$59,174	$63,827	$56,141
Length of Stay (days)	3.47	3.37	3.97
Chronic Conditions	7.58	7.07	7.64

Urban Teaching Vs. Urban Non-Teaching

When comparing urban hospitals by teaching status, there was no significant difference in LOS or average age of admission. When total costs and chronic conditions are examined, a counterintuitive trend is noted. For urban teaching hospitals, the number of chronic conditions was significantly higher (p<0.001) when compared to non-teaching hospitals. However, when total charges are compared, the data shows urban teaching hospitals costs are lower (p=0.016).

Urban Teaching Vs. Rural

Rural hospitals had longer lengths of stay when compared to urban teaching hospitals (p=0.015). There was no difference between cost (p=0.336), age of admission (p=0.792), and number of chronic conditions (p=0.810) between urban teaching and rural hospitals.

Urban Non-Teaching Vs. Rural

Between urban non-teaching and rural hospitals, there was no difference between age of admission (p=0.334). Rural hospitals were associated with higher numbers of chronic conditions (p=0.010) and longer lengths of stay (p=0.0093). Despite this, total costs were significantly lower (p=0.013) as compared to urban non-teaching hospitals.

## Discussion

Total hip arthroplasty (THA) is among the most common orthopedic surgical procedures performed in the United States and demand has risen dramatically over the last two decades, with estimates continuing to rise. One reason for THA being so common is that it drastically improves patient quality of life and function. Although largely beneficial to the patients, there are also notable associated risks with undergoing THA. Chronic obstructive pulmonary disease (COPD) is a major risk factor when undergoing any major surgical procedure, including THA. COPD has been found to be one of the most serious comorbidities in patients with osteoporosis, which is a common cause of THA [12]. With the potential for such serious complications, the connection between COPD and THA is imperative to both physicians and patients.

Consistent with the studies by Liao et al. and Yakubek et al., our study showed that patients with COPD had significantly higher mortality rates and length of stay (LOS) as compared to the non-COPD group [8,13]. Our goal, however, was to compare the differences between the results of THA in patients with COPD among teaching and non-teaching hospitals. Our data suggest that teaching hospitals have decreased costs for patients with COPD that undergo THA while also having a greater mean number of chronic conditions per patient. The natural assumption is that the teaching hospitals' spend is associated with higher costs. However, based on our data, this thought process is flawed in certain cases. The results of our study suggest that teaching hospitals are able to optimize care and be more efficient, which leads to lower costs to the patient. A study performed by Kowalik et al. showed that primary THA performed at teaching hospitals cost, on average, $47,514 and $49,445 at non-teaching hospitals and revision THA performed at teaching hospitals cost, on average, $67,820 and $66,990 at non-teaching hospitals [14]. COPD was not studied as a complicating comorbidity, but the higher cost of revision surgery in teaching hospitals may be due to an increased incidence of COPD among revision patients. The presence of resident physicians also leads to the potential of surgery with state-of-the-art procedures and equipment, which can decrease risk factors and cost. Likewise, rural hospitals, which are often assumed to have fewer resources, seem to be the most efficient at managing costs with regards to COPD patients undergoing THA.

Limitations to our study include the fact that teaching hospitals may or may not have residency programs with residents involved in the patient’s direct THA or COPD care. In addition, hospitals within the teaching or non-teaching cohorts can have a wide array of services, surgical techniques, and levels of expertise from one individual facility to the next. Our sample size may not be large enough, especially for rural hospitals, to be adequately representative of the national norm.

## Conclusions

The current study identified that, in contrast to previous studies, teaching hospitals have lower costs of care when treating patients undergoing THA with concurrent COPD when compared to urban non-teaching hospitals. This data suggest that teaching hospitals and rural hospitals are able to utilize healthcare in a cost-effective way, although the specific mechanisms of how teaching status affects costs are undoubtedly multifactorial. Likewise, despite longer lengths of stay and increased numbers of chronic conditions, rural hospitals have the lowest costs of all. Using this data, researchers can further examine the individual mechanisms and help diminish disparities among rural, teaching, and non-teaching hospitals. Our results may also aid surgeons and primary care providers in hospital selection or recommendations when considering THA in patients with COPD.
